# Comparison of Pressurized Water Extraction With Ultrasound Assisted Extraction for Isolation of Phycobiliproteins From *Arthrospira platensis* (Spirulina)

**DOI:** 10.1002/pca.3486

**Published:** 2024-12-10

**Authors:** Lenka Burdějová, Pavlína Dadajová, Barbora Kudláčková, Filip Duša

**Affiliations:** ^1^ Institute of Analytical Chemistry of the Czech Academy of Sciences Brno Czech Republic; ^2^ Department of Chemistry, Faculty of Science Masaryk University Brno Czech Republic

**Keywords:** *Arthrospira platensis*, phycocyanin, pressurized water extraction, spirulina, ultrasound assisted extraction

## Abstract

**Introduction:**

Cyanobacterium *Arthrospira platensis* (AP) (Nordstedt) Gomont contains high content of phycobiliproteins (PBP), which are an important source for food industry. Methods effectively extracting proteins contained in AP cells are demanded to provide a supply of the material. Water‐based extraction methods are advisable due to the high solubility of the PBP.

**Objectives:**

Extraction techniques such as ultrasound assisted extraction (UAE) and pressurized water extraction (PWE) are popular due to their environmental friendliness, better extraction efficiency, and faster extraction process. In this paper, efficiency of the two methods is compared.

**Materials and Methods:**

PWE along with UAE is utilized for release of PBP from the AP cells. The extraction parameters including time, temperature, pressure, and ultrasound intensity are tested to obtain the most efficient setup. The methods were evaluated using sodium dodecyl sulfate polyacrylamide gel electrophoresis (SDS‐PAGE), and the replicates of PWE extracts were further analyzed by capillary isoelectric focusing with laser‐induced fluorescence (cIEF‐LIF).

**Results:**

The developed PWE method using higher pressure treatment at lower temperature was significantly faster than UAE methods, and the SDS‐PAGE results showed a high content of phycobiliproteins in the extracts. cIEF‐LIF analysis showed that the sequential PWE of individual samples was repeatable, and the mild extraction provided a fluorescent profile similar to the commercially available C‐phycocyanin standard.

**Conclusion:**

Pressurized water extraction was shown to be an efficient, rapid, and well‐automated extraction method for AP proteins in general, including bioactive phycobiliproteins. Obtained results encourage the use of PWE in small‐scale analytical applications for primary extraction of proteins.

## 1 Introduction

Phycobiliproteins (PBP), water‐soluble photosynthetic protein‐pigment complexes found in cyanobacterium *Arthrospira platensis* (AP) (Nordstedt) Gomont, commonly known as Spirulina, are valuable natural products with beneficial health effects, including antioxidant, anti‐inflammatory, neuroprotective, anticancer, and immunomodulatory activities [1, 2, 3, 4]. They are located on the surface of the thylakoid membranes as large rounded granules called phycobilisomes. Phycobilisomes consist of allophycocyanin (APC) cores peripherally surrounded by chlorophycocyanin (C‐PC) rods. Twenty percent of the dry weight of spirulina consists of C‐PC, and it is the major PBP [5, 6].

Due to their interesting functional properties, they have various applications in the food, cosmetic and pharmaceutical industries, as well as in medical diagnostics, immunology, bioengineering, biomedical, and analytical research [7, 8, 9]. PBPs are widely used as dietary supplements, natural food and cosmetic colorants, protein markers for electrophoresis, fluorescent reagents, probes and tracers with applications in flow cytometry, fluorescent immunoassays, fluorescence microscopy, and as a substitute for ethidium bromide for immunological analysis and DNA staining [7, 10, 11, 12] and as a photosensitizer in photodynamic therapy for cancer treatment [13, 14].

PBP can be extracted from AP with water using various conventional extraction approaches such as maceration, freeze‐thawing, French press, direct osmosis, and lysozyme digestion [5, 9, 15, 16, 17, 18, 19]. However, the main limitations of conventional extraction methods are moderate extraction efficiency, long extraction times, and the possibility of disruption or denaturation of PBP. Modern green extraction techniques such as ultrasound‐assisted extraction (UAE), high‐pressure homogenization (HPH), pressurized water extraction (PWE), and supercritical carbon‐dioxide extraction between others eliminate these problems and become popular alternatives due to their environmental friendliness, better extraction efficiency, and faster extraction process [20, 21].

UAE, which uses ultrasound for particle disruption, is one of the most widely used green extraction techniques for PBP extraction due to its simplicity and general availability of equipment such as the ultrasonic baths and probes [22, 23, 24, 25, 26]. In recent years, there is a number of works that apply pressures up to 500 MPa for PBP isolation [27, 28], suggesting the potential of using high pressures for extractions. However, the use of an automated extraction method using pressurized water at low temperatures and short times for the isolation of PBP from AP has not been extensively investigated so far. Zhou et al. [29] in their study concluded that the increased amount of small molecular weight proteins in the pressurized liquid extract (10 MPa) was due to the increase of their solubility. Damage to the primary structure of high molecular weight proteins was not observed even when using high pressure food processors at pressures above 150 MPa. Below this limit, only a change in the quaternary structure of pressurized proteins was observed. Therefore, in this study, we present the environmentally friendly PWE rapid method for PBP extraction from AP and compare it with UAE‐based methods using two different extraction devices (ultrasonic bath, probe). Extraction methods (PWE or UAE) in combination with sodium dodecyl sulfate polyacrylamide gel electrophoresis (SDS‐PAGE) were carried out to evaluate the extraction efficiency of each method. Additionally, capillary isoelectric focusing analysis with laser‐induced fluorescence detection (cIEF‐LIF) was performed to examine the extracted PBP.

## 2 Materials and Methods

### 2.1 Chemicals and *Arthrospira platensis* Material

Analytical or gradient purity chemicals were used during the study. 2‐sulfanylethanol, 2‐aminoethanoic acid (glycine), N,N,N′,N′‐tetramethyl ethylenediamine (TEMED), protein wide range molecular weight (WRMW) standard marker (6.5–200 kDa), L‐arginine, iminodiacetic acid, lysine, urea, H_3_PO_4_, NaOH, and the C‐phycocyanin were purchased from Sigma‐Aldrich (St. Louis, MO, USA), propan‐2‐ol was provided from VWR International (Radnor, PA, USA), acetic acid was provided by Penta (Prague, Czech Republic), sodium dodecyl sulfate (SDS) was purchased from J.T. Baker (Deventer, Netherlands), Rotiphorese®Gel 30 (37.5:1) was purchased from Carl Roth (Karlsruhe, Germany), ammonium persulfate (APS) was purchased from Bio‐Rad Laboratories (Hercules, CA, USA), Laemmli buffer and PageBlue™ Staining Solution were purchased from Thermo Fisher Scientific (Waltham, MA, USA), and the carrier ampholyte solution Pharmalyte 3–10 (36% w/v), Sinulyte (40% w/v), and cIEF gel were purchased from AB Sciex (AB Sciex LLC, Framingham, MA, USA). The fluorescein‐based p*I* markers used for cIEF were synthesized in‐labor in our laboratory [30]. The water used in all experiments was purified with a Milli‐Q A10 Gradient (Millipore, Burlington, USA). *Arthrospira platensis* powder, of Chinese origin produced by MANU JTC s.r.o., Ludgeřovice, Czech Republic, was used for the preparation of crude protein extracts. AP powder was stored under dry and dark conditions.

### 2.2 Extraction Methods Used for Isolation of *Arthrospira platensis* Phycobiliproteins

Three protein extraction protocols were compared, that is, PWE, UAE using ultrasonic bath, and UAE using homogenizer probe, to determine the most suitable extraction method for primary extraction of AP proteins mainly phycobiliproteins.

#### 2.2.1 Pressurized Water Extraction Using Pressurized Extractor

The crude protein samples were prepared on the in‐lab‐constructed pressurized extractor working in a static mode, as previously described in detail by our group [31]. Fifty milligrams of AP fine powder was mixed with 13.5 g of 500 μm diameter glass beads. The mixture was packed into an 11 mL stainless steel extraction cell and extracted at a constant pressure of 15 MPa. Extraction parameters such as temperature, number of extraction cycles, and time were standardized.

In the first step, the extraction temperature was tested in the range of 40°C to 80°C with 10°C step and 5 min extraction time. In the next step, one to five extraction cycles were tested at the optimized temperature each lasting 5 min. Finally, extraction times of 2, 5, 10, 20, and 30 min were tested at the optimized conditions. Fresh extraction solvent was used for each extraction cycle, and each extraction was followed by a 2‐min purging with nitrogen. The collected extracts were cooled down to room temperature (25 ± 2°C) and processed as described below. All extractions were performed in triplicates.

#### 2.2.2 Ultrasound Assisted Extraction Using Ultrasonic Bath

The protein crude samples were prepared according to following extraction procedure: 50 mg of AP fine powder was suspended in 11 mL of deionized water and sonicated in 5‐, 15‐, 30‐, 45‐, 60‐, and 90‐min intervals using ultrasound bath Sonorex Digital 10P Badelin (Labicom s.r.o., Prague, Czech Republic) and frequency of 35 kHz. The temperature was kept below 35°C. Finally, the mixture was centrifuged for 5 min using laboratory centrifuge EBA 20 (Hettich, Tuttlingen, Germany) at 3461 g (6,000 rpm, 15 mL falcon tubes) and 25°C. The resulting supernatant was removed into new tube and used for further analysis. All extractions were performed in triplicate.

#### 2.2.3 Ultrasound Assisted Extraction Using Ultrasonic Homogenizer Probe

Crude protein samples were prepared using SONOPULS MS 72 ultrasonic homogenizer probe (Bandelin Ultraschall Apparatebau GmbH, Berlin Germany), which was connected to a Bandelin GM 3100 sonicator. A 15 mL conical centrifuge test tube was filled with hydrated AP powder (10 mg of fine powder in 2.2 mL of deionized water) and fixed on a stand inside a soundproof box. The probe was immersed in the sample suspension so that there was no contact between the probe and the bottom or walls of the tube. The top of the tube was sealed with a parafilm foil to prevent escape of the sonicated liquid. The tube was further immersed in a beaker of water containing ice to prevent overheating of the sample. The ice‐water mixture was changed after each sonication run.

Different sonication parameters affecting the extraction of phycobiliproteins such as total sonication time and sonication pulse ‘on and off’ time cycles were optimized. Experimental series were carried out: Series 1: amplitude: 97%, ultrasonic pulse on/off time cycles 15/15 s, total sonication time: 1, 2, 3, 4, 5, and 10 min, and Series 2: amplitude: 97%, sonication pulse on/off time cycles 1/1, 5/5 and 15/15 s, total sonication time: 4 min. Sonicated samples were centrifuged for 5 min at 3461 g and 25°C, and the supernatant was collected for further analysis. All extractions were performed in triplicate.

### 2.3 Polyacrylamide gel Electrophoresis of Different *Arthrospira platensis* Extracts

Gel electrophoresis was performed using the Mini‐PROTEAN Tetra cell system (Bio‐Rad Laboratories). The sodium dodecyl sulfate‐polyacrylamide gel electrophoresis (SDS‐PAGE) mini gels were prepared in our laboratory. The 15% separation gel solution for the preparation of two mini gels with size 10 cm × 8 cm × 1 mm contained 5.63 mL of acrylamide‐bisacrylamide solution—Rotiphorese gel 30 (37.5:1), 5.63 mL of 0.75 mol L^−1^ Tris–HCl buffer pH 8.8, 112.5 μL of 10% (w/v) SDS, 9 μL of TEMED, and 60 μL of 10% (w/v) APS. Immediately after mixing the gel components, the gel solution was poured into the gel casting assembly, and then, the gel solution was overlaid with 1 mL of 2‐methylpropan‐1‐ol/water solution (10:1) and was allowed to polymerize for 1 h. After 1 h, the organic layer was removed and replaced with a 4% stacking gel. The solution for two mini gels contained 1 mL of acrylamide‐bisacrylamide solution, 5 mL of 0.25 mol L^−1^ Tris–HCl buffer pH 6.8, 100 μL of 10% (w/v) SDS, 3.8 mL of deionized water, 5 μL of TEMED, and 150 μL of 10% (w/v) APS. The gel was allowed to polymerize for 1 h, then removed from the casting assembly, and used for electrophoresis.

The final extract prepared by different extraction methods (see Sections 2.2.1, 2.2.3) was mixed with commercial Laemmli sample buffer in a ratio of 1:1, vortexed and centrifuged using laboratory centrifuge Eppendorf MiniSpin plus (Eppendorf, Hamburg, Germany) at 14,000 g, 25°C for 1 min. The sample was then heated in boiling water bath for 5 min. Five microliters of WRMW standard marker or 10 μL of a sample was loaded per each well. The gels were run in lab‐made Tris‐Glycine buffer (25 mmol L^−1^ Tris, 192 mmol L^−1^ glycine, 0.1% (w/v) SDS pH 8.3) at constant voltage 160 V for 1 h. After the run, the gels were fixed with 25% (v/v) propan‐2‐ol and 10% (v/v) acetic acid fixing solution for 15 min, stained with PageBlue™ Protein Staining Solution for 6 h, and the background color was destained with deionized water for 3 h.

### 2.4 cIEF‐LIF Analysis of Different *Arthrospira platensis* Extracts

The cIEF‐LIF separation was performed on the P/ACE™ MDQ plus instrument. Measurements were performed using 50 μm ID capillary with neutral linear polyacrylamide coating and length 20/30.2 cm (*l*_*eff*_/*l*_*tot*_). The temperature of the sample storage compartment was maintained at 10°C, and the capillary was tempered at 20°C. The LIF module was used for detection, which included a solid state 488 nm laser as an excitation source and a detector with two simultaneous detection channels with 50/50 beam splitter. The first channel contained a notch filter 488 nm followed by a bandpass filter λ_em_ 520/20 nm and the second channel contained the notch filter 488 nm followed by a longpass filter λ_em_ 550 nm. Data were recorded and processed by 32 Karat software (AB Sciex LLC, Framingham, MA, USA).

One‐hundred microliters of eight replicas of crude PWE extracts (extracted at 40°C, 2‐min cycle, 15 MPa) were lyophilized and stored in a freezer at −20°C. For the cIEF analysis, the extracts were dissolved in 200 μL of deionized water and diluted 25‐fold in the sample master mix, which consisted of 2.4 mol L^−1^ urea, 80% v/v cIEF gel, 1.92% w/v background ampholytes (Pharmalyte 3–10), 60 mmol L^−1^ cathodic stabilizer (L‐arginine), 1.6 mmol L^−1^ anodic stabilizer (iminodiacetic acid), and/or four fluorescent p*I* markers (p*I* 4.05, 5.61, 6.76, and 8.73 at concentration 11–0.14 ng mL^−1^) [30]. For the C‐phycocyanin standard, a concentration of 40 μg mL^−1^ was used.

The cIEF method was performed according to the standard protocol developed by Sciex [32]. The capillary was first rinsed with urea for 2 min at 3.4 bar, then with water for 1.5 min at 3.4 bar, followed by sample injection for 99 s at 3.4 bar. A solution of 200 mmol L^−1^ H_3_PO_4_ was used as anolyte, and 300 mmol L^−1^ NaOH was used as catholyte. During the focusing step, voltage of 25 kV was applied for 15 min. Chemical mobilization was performed at 30 kV for 30 min with 350 mmol L^−1^ CH_3_COOH. At the end of the run, the capillary was rinsed with water at 3.4 bar for 1.5 min. The p*I*s of the main peaks were determined using fluorescein‐based low‐molecular‐mass p*I* markers, and the obtained electropherograms were compared with the commercial C‐PC standard.

## 3 Results and Discussion

The selection of the most appropriate protein extraction method is an important step to obtain the desired compounds from AP material. The present study focuses on the comparison of three green extraction protocols in order to select the most efficient and rapid method for the primary isolation of phycobiliproteins.

PWE was compared with UAE‐based methods using two different ultrasonic devices: ultrasonic bath and homogenizer probe. In all cases, water was used as the extraction solvent due to the fact that phycobiliproteins are well soluble in water and no further purification steps are required during the protein isolation. The extraction efficiency of the methods was evaluated by SDS‐PAGE and cIEF. PBPs are large oligomers consisting of smaller subunits typically about 170 AA (amino acids) in length or 20 kDa in size. Therefore, after being subjected to the denaturing conditions of SDS‐PAGE, the PBPs disassemble and smaller proteins around 20 kDa, representing the individual PBP subunits, could be observed on the stained gel [33, 34].

**FIGURE 1:**
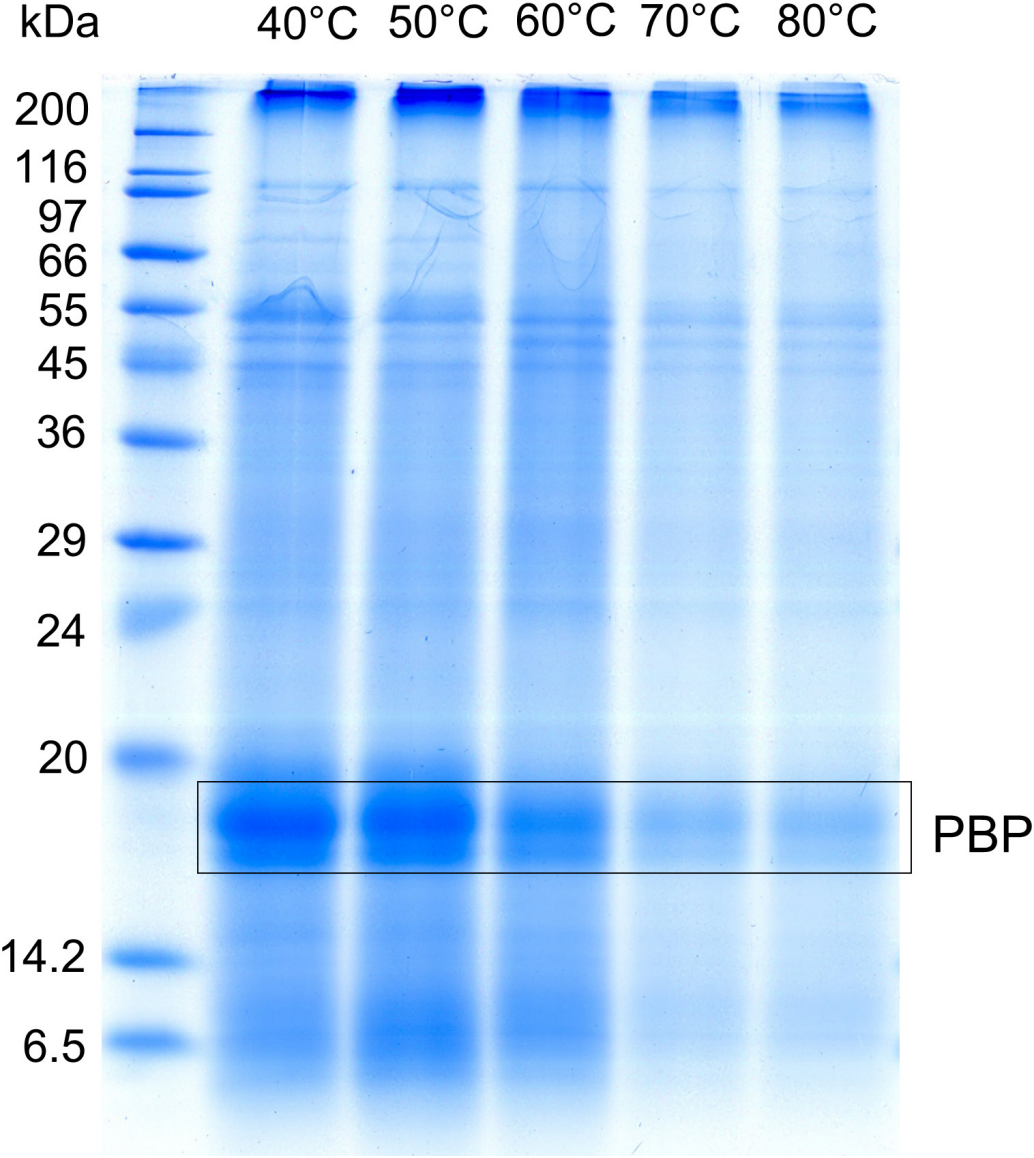
Determination of the optimal pressurized water extraction (PWE) temperature for the isolation of *Arthrospira platensis* phycobiliproteins (PBP). Optimized temperature range from 40°C to 80°C with 10°C steps, extraction time 5 min and pressure 15 MPa.

PWE parameters such as temperature, number of extraction cycles, and time, which are the most commonly considered parameters, were optimized for the recovery of AP proteins including bioactive PBP. Temperature as the main parameter affecting the extraction rate and efficiency, as well as the physicochemical properties of the proteins, was optimized in the range of 40°C to 80°C with a 10°C step and 5 min extraction time at a constant pressure of 15 MPa. Increasing the extraction temperature resulted in a gradual decrease in protein yields (Figure 1), indicating that higher temperatures induce protein degradation. The optimal temperature for the extraction of AP proteins including PBP (marked with a frame in Figure 1) was 40°C, which is also the minimum recommended temperature with the PWE instrument. Previous study by Zhou et al. [29] confirmed that 40°C is better than 25°C for extraction of PBP. Our results are in contrast with the study of Carlos et al. [35], which recommended 25°C with very long extraction times (360 min) for PBP isolation.

**FIGURE 2:**
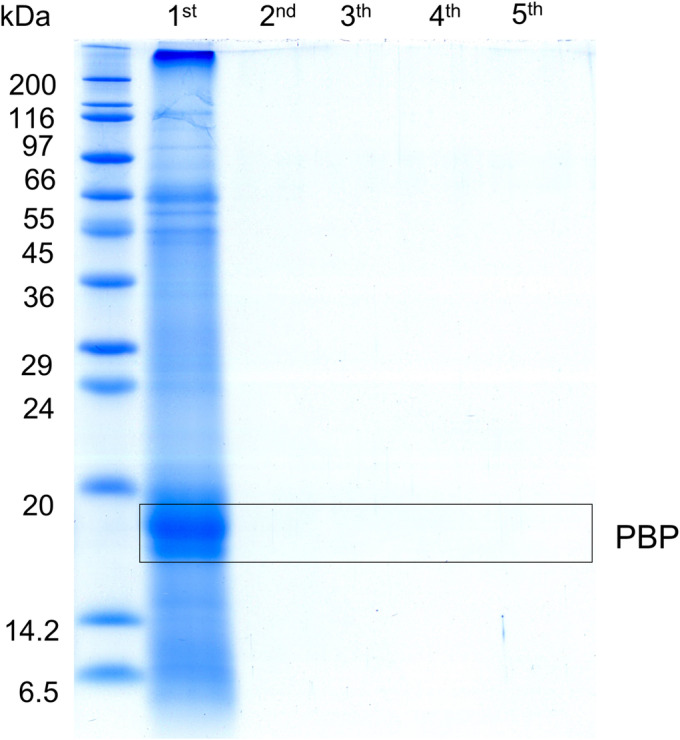
Determination of the optimal number of pressurized water extraction (PWE) cycles for the isolation phycobiliproteins (PBP) from *Arthrospira platensis*. Optimized number of extraction cycles from 1 to 5, duration of each cycle 5 min at 40°C and pressure of 15 MPa.

The number of extraction cycles (from one to five) was another important parameter to be tested. In this experiment, the extraction temperature was 40°C, pressure was 15 MPa, and each cycle lasted 5 min. Fresh solvent (water) was used for each extraction cycle. The extracts from each particular cycle were collected and analyzed separately. The results in Figure 2 suggest that one cycle of 5 min was sufficient for complete extraction of proteins. The additional cycles did not extract any more proteins detected by SDS‐PAGE.

**FIGURE 3:**
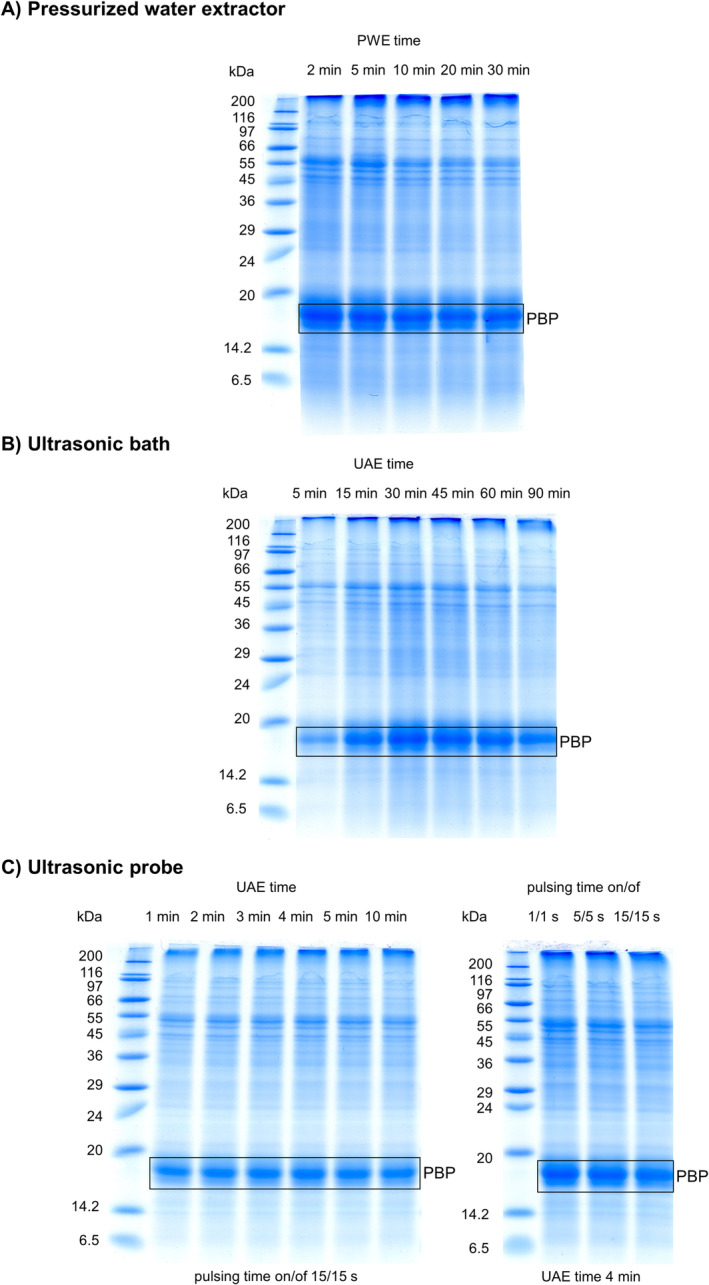
Comparison of SDS‐PAGE separations of *Arthrospira platensis* proteins including phycobiliproteins (PBP) extracted with (A) pressurized water extractor (extraction time 2, 5, 10, 20, and 30 min, one cycle, at pressure 15 MPa) with (B) ultrasonic bath (extraction time 5, 15, 30, 45, 60, and 90 min, frequency of 35 kHz and 35°C), and with (C) ultrasonic probe (Series 1: extraction time 1, 2, 3, 4, 5, and 10 min, amplitude 97% and pulse on/off time cycles 15/15 s, Series 2: extraction time 4 min, amplitude: 97%, ultrasonication pulse on/off time cycles 1/1, 5/5, and 15/15 s).

In the next step, the effect of extraction cycle length was studied under the optimized conditions: temperature of 40°C, one extraction cycle, and the constant pressure of 15 MPa. Five different extraction times were tested: 2, 5, 10, 20, and 30 min. Extraction time is a necessary parameter to ensure the complete extraction of the AP proteins. Typically, it is the time at which the equilibrium between the solvent and the extracted compounds is established. This parameter allows to select the shortest extraction time and thus to reduce the operating costs of the extraction process. The *extraction time* affects both the *extraction* efficiency and the degree of protein degradation [36]. Our results show that the shortest extraction cycle time tested (2 min) was sufficient for complete extraction of AP proteins (Figure 3A). Prolonging of the time up to 30 min showed a slight decrease in the content of larger proteins with a molecular weight in the range of 29–116 kDa. No such effect was observed for PBP with its relatively low molecular weight of 16–18 kDa. Overall, the results suggest that the long extraction time may induce protein degradation and does not increase the extraction yield of PWE for the most AP proteins. The final optimal PWE conditions for AP proteins were a 2‐min extraction cycle at a pressure of 15 MPa and a temperature of 40°C. Compared to previous studies by Herrero et al. [37] and Carlos et al. [35], which tested PWE of AP at 25°C, the total extraction time was reduced dramatically from 75 min, respectively, 360 to 2 min, when PWE of AP at 40°C was used.

Finally, the optimized PWE method was compared with two ultrasound assisted methods using ultrasonic bath and homogenizer probe. In the case of UAE using a bath, fine AP powder was mixed with water in the same ratio as used for PWE and sonicated in an ultrasonic bath at temperature kept below 35°C and a frequency of 35 kHz for 5, 15, 30, 45, 60, and 90 min. The results (Figure 3B) demonstrated that UAE time shorter than 30 min was insufficient for AP protein isolation. Maximum protein yield was obtained after 30 min of sonication. For longer sonication times, a decrease in protein yield with increasing extraction time was observed. Considering the extraction time, PWE is clearly the faster extraction method. Moreover, the sonication bath needs cooling to remove heat generated during sonication, which can be problematic with regular sonication tank designs. Furthermore, the precise position and immersion depth of the extraction vessel must be maintained to avoid variations in UAE efficiency due to uneven intensity distribution within the sonication bath (based on sonication source position, input power, and frequency) [38].

In the case of UAE probe sonication, fine AP powder was mixed with water in the same ratio as for PWE, and it was sonicated in 15‐mL conical test tubes using an ultrasonic homogenizer probe at an amplitude of 97% and ultrasonication pulse on/off time cycles of 15/15 s for durations of 1, 2, 3, 4, 5, and 10 min. The results showed (Figure 3C) that all extraction times tested were able to extract proteins from AP. However, the 4‐min UAE time showed the highest protein yield. When the UAE time was increased from 5 to 10 min, a slight decrease in extraction efficiency was observed for the PBP fractions. Therefore, 4‐min extraction was selected as optimal for probe UAE. In the next step, 4‐min UAE extraction time was tested at 97% amplitude and different ultrasonic pulse on/off time cycles especially 1/1, 5/5, and 15/15 s. It was found that 1‐s pulse at 97% amplitude and 1‐s cooldown intervals released the highest amount of AP proteins, mainly PBP. At the same time, more bands were identified in the extracts treated with ultrasonic pulse on/off time cycles of 1/1 s compared to other conditions tested. The results suggested that the optimal conditions for extraction using ultrasonic probe were extraction time 4 min, amplitude 97%, and on/off time cycles 1/1 s. In comparison with PWE, UAE with probe was slightly slower; it needed 4 min for efficient extraction of the same amount of PBP than PWE method. From the feasibility point of view, the UAE using probe seems to be a less suitable method than PWE due to the necessary optimization of ultrasonic on/off cycles, the need to externally cool the sample during extraction, and the difficulty of using larger sample amounts and solvent volumes due to the need of larger containers to prevent extract escape. In addition, local overheating of the sample could occur during UAE using probe, which may have caused inadvertent breakage of longer protein chains during intensive sonication [24]. Furthermore, UAE requires additional sample handling such as centrifugation and filtration. In contrast, filtration is already included in PWE as the extracts are filtered through a frit into a collection vial.

**FIGURE 4:**
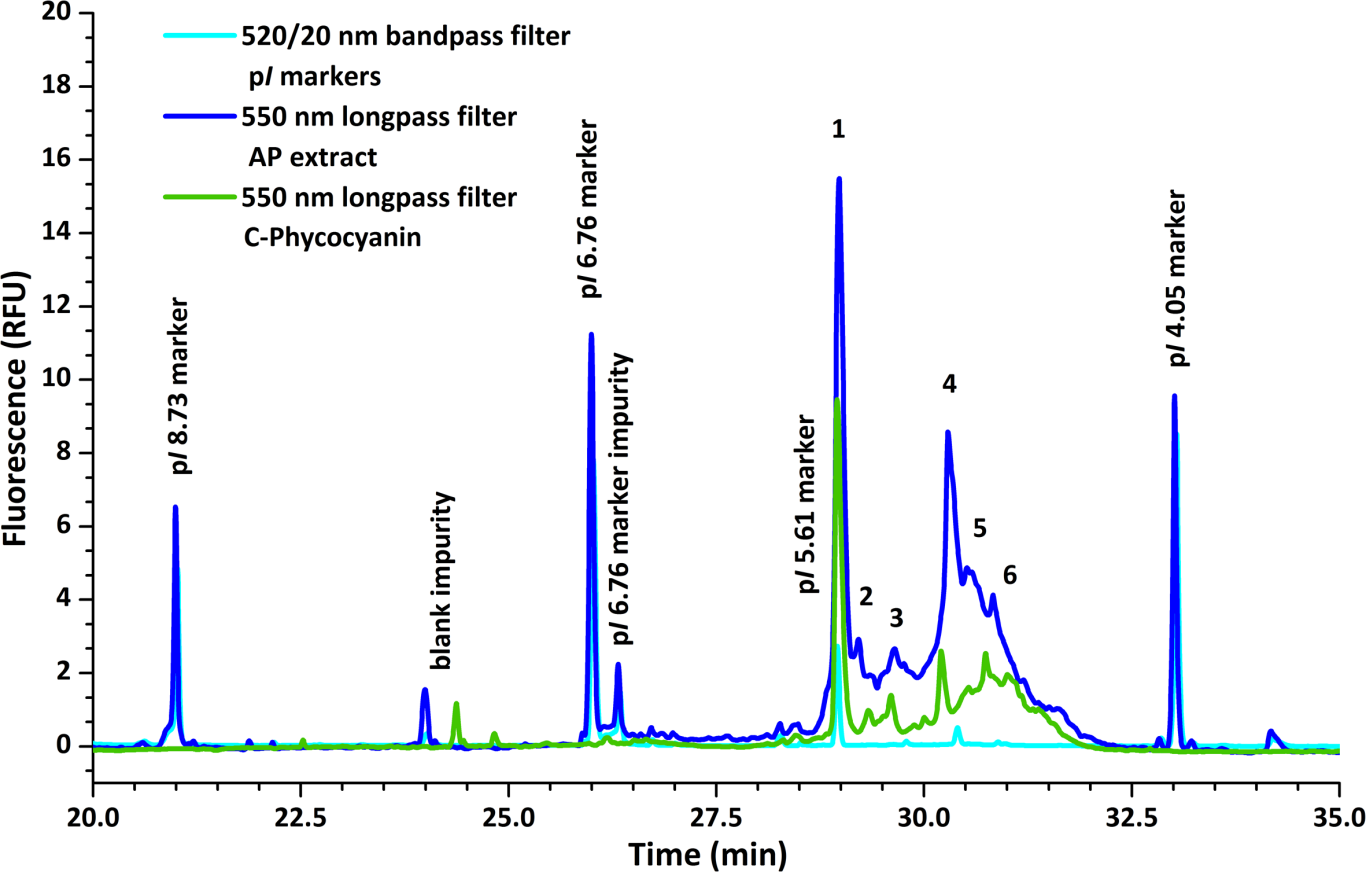
Representative cIEF‐LIF separation of the *Arthrospira platensis* (AP) extract overlaid with C‐phycocyanin standard, containing fluorescent p*I* markers 4.05, 5.61, 6.76, and 8.73. The six most prominent areas marked in the figure are characterized in Table 1. Electrophoretic conditions are described in Section 2.4.

Finally, cIEF‐LIF analysis was performed on the PWE extracts. The intrinsic natural fluorescence of PBPs is mediated by a phycobilin cofactor in their structure. Phycocyanobilin present in the protein C‐phycocyanin, which is specific for AP, is characterized by emission of fluorescence at wavelengths of 540 nm and above (**λ**_max_ 640 nm) [39]. It has been reported that urea concentrations up to 5 mol L^−1^ have no effect on protein unfolding [40] and should not cause loss of fluorescence, which was confirmed by cIEF analysis using 2.4 mol L^−1^ urea in the sample mixture. C‐PC in solution has also been reported to exist in the forms of monomer, trimer, hexamer, and higher aggregates. However, at pH 4.5–6.0 and moderate ionic strength, C‐PC exists in a monomer–trimer system [41]. cIEF analysis of a C‐PC standard showed multiple peaks in the electropherogram in the region from 4.87 to 5.61 (Figure 4). Six main peaks were selected within the peak cluster, and their p*I*s were calibrated (Table 1).

**TABLE 1:** Determined p*I*s and average percent area distribution of the most prominent peaks observed on the electropherograms obtained from the *Arthrospira platensis* extracts (*n* = 8) in comparison with C‐phycocyanin.

Peak #	1	2	3	4	5	6
**C‐phycocyanin standard**						
p*I*	5.61	5.40	5.20	5.09	4.96	4.87
Area (%)	25.1	10.21	4.93	8.50	7.74	15.67
***Arthrospira platensis* extract (*n* = 8)**						
p*I*	5.61	5.49	5.36	5.10	5.02	4.90
p*I* SD	0.02	0.02	0.01	0.02	0.02	0.02
Area (%)	24.41	7.83	11.95	23.27	12.20	13.21
Area RSD (%)	22.96	7.84	10.23	10.03	29.57	23.25

**FIGURE 5:**
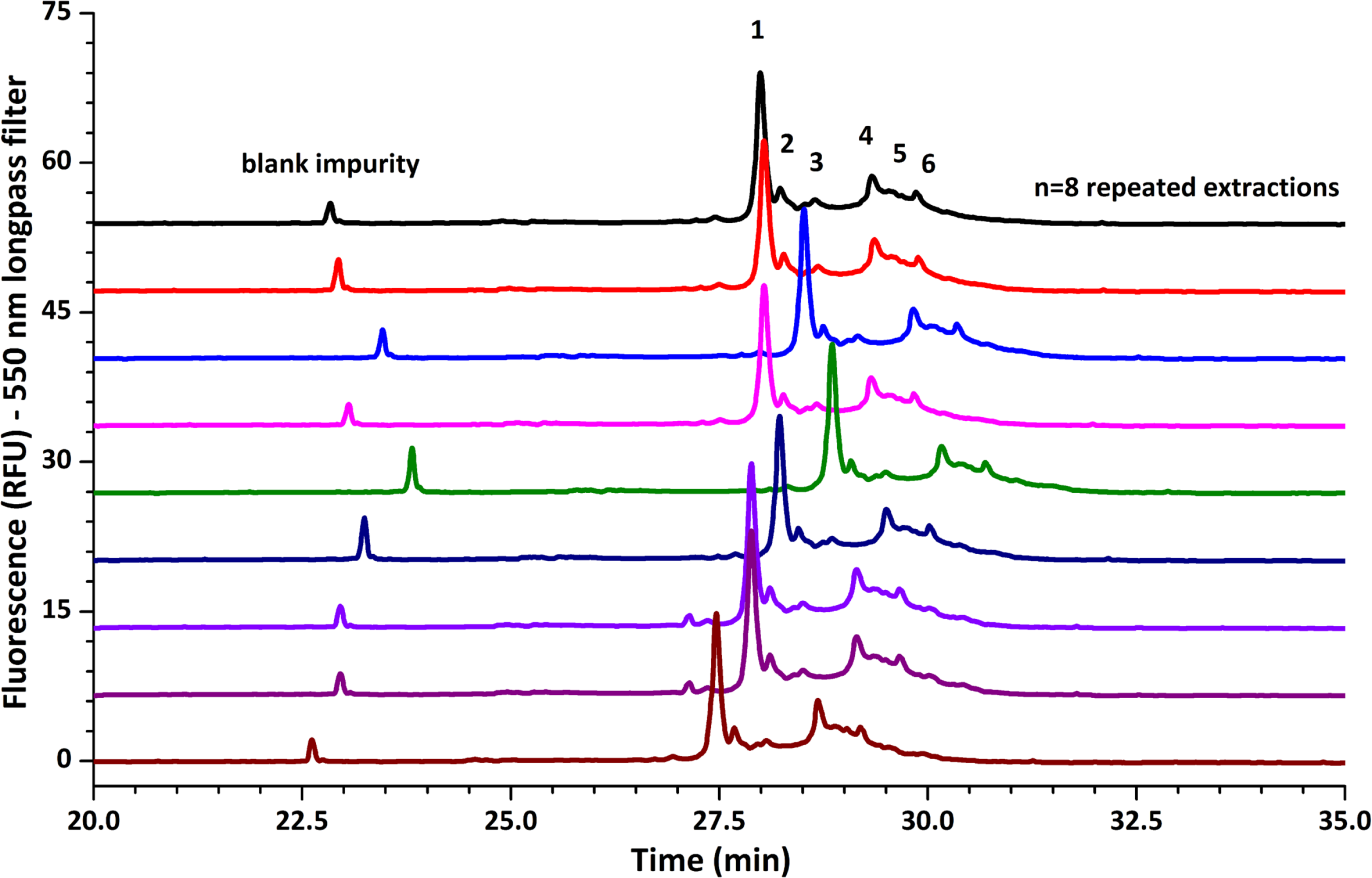
Repeatability and fluorescence intensity of replicate *Arthrospira platensis* extracts (*n* = 8) obtained by the optimized PWE method (40°C, 2 min, 15 MPa, one cycle). Electrophoretic conditions are described in Section 2.4.

Eight crude AP PWE extracts were separated by cIEF‐LIF and the extraction efficiency and repeatability of the optimized PWE method were evaluated. The PWE extract peaks span from p *I* 4.90 to 5.61 (Figure 5). The obtained peak profile was compared with a C‐PC standard (Figure 4). Six peaks from the cluster aligned accurately with electropherogram of the C‐PC standard. The p*I* values of the selected peaks were determined using the fluorescent p*I* markers. Furthermore, the peak areas were evaluated as the area under the curve with vertical splitting lines in the peak cluster. The values obtained, together with the standard deviations, are shown in the Table 1. The p*I* values of the individual peaks fluctuated on the level of 0.02 pH units, confirming that the same proteins were present in the extracts. In addition, the peak pattern was very stable and uniform for all the extracts analyzed, suggesting a repeatable extraction process. The total fluorescence peak area of eight consecutive extracts varied with a relative standard deviation of 14.3%. The obtained cIEF‐LIF data show that the optimized PWE method was able to extract C‐PC from the AP cells in a repeatable manner, maintaining its conformation and natural fluorescence. The results showed that the sequential PWE of individual Spirulina samples was repeatable and the mild extraction provided the fluorescent p*I* profile similar to the commercially available C‐phycocyanin standard. Collectively, the results obtained encourage the use of PWE in small‐scale analytical applications for primary extraction of proteins, including phycobiliproteins.

## Data Availability

Data will be made available on request.
